# A comparison of unamplified and massively multiplexed PCR amplification for murine antibody repertoire sequencing

**DOI:** 10.1096/fba.1017

**Published:** 2018-10-25

**Authors:** Trisha A. Rettig, Michael J. Pecaut, Stephen K. Chapes

**Affiliations:** ^1^ Division of Biology Kansas State University Manhattan Kansas; ^2^ Division of Biomedical Engineering Sciences (BMES) Loma Linda University Loma Linda California

**Keywords:** heavy chain, high‐throughput sequencing, immunoglobulin genes, mouse, repSEQ

## Abstract

Sequencing antibody repertoires has steadily become cheaper and easier. Sequencing methods usually rely on some form of amplification, often a massively multiplexed PCR prior to sequencing. To eliminate potential biases and create a data set that could be used for other studies, our laboratory compared unamplified sequencing results from the splenic heavy‐chain repertoire in the mouse to those processed through two commercial applications. We also compared the use of mRNA vs total RNA, reverse transcriptase, and primer usage for cDNA synthesis and submission. The use of mRNA for cDNA synthesis resulted in higher read counts but reverse transcriptase and primer usage had no statistical effects on read count. Although most of the amplified data sets contained more antibody reads than the unamplified data set, we detected more unique variable (V)‐gene segments in the unamplified data set. Although unique CDR3 detection was much lower in the unamplified data set, RNASeq detected 98% of the high‐frequency CDR3s. We have shown that unamplified profiling of the antibody repertoire is possible, detects more V‐gene segments, and detects high‐frequency clones in the repertoire.

**Abbreviations**
AMV: Avian Myeloblastosis Virus
D: diversity gene segment
IMGT: ImMunoGeneTics
J: joining gene segment
MMLV: Moloney Murine Leukemia Virus
TRNA: total RNA
V: variable gene segment

## 1 INTRODUCTION

Antibodies are B‐cell proteins that play a vital role in adaptive immunity. These complex molecules, and their diverse specificities are defined at several levels. At the protein level, these molecules are heterodimers joined with disulfide bonds between heavy and light chains. At the genetic level, the antibody specificity is influenced by semi‐randomly recombining a variable (V), diversity (D), and joining (J) gene segments that are encoded in the genome. This V‐D‐J, or in the case of the light chain, V‐J rearrangement, also influences antibody specificity by random and semi‐random base insertions or deletions during the recombination process. In the end, the collection of antibodies produced, the antibody repertoire, is a fingerprint of what antigens an organism has been exposed to and a measure of immunocompetence. Sequencing has become easier, cheaper, and faster in recent years. Antibody repertoires have been sequenced in numerous species, in response to vaccinations^1^, ^2^, ^3^ and infections,^4^, ^5^, ^6^ and have been employed in cancer detection providing valuable feedback regarding the immune system's response to challenges and for early cancer detection in patients.^7^, ^8^

In an effort to supplement unamplified MiSeq data sets used in our laboratory,^9^ we explored the use of commercial processes that use technologies to amplify sequences to increase the depth of coverage of specifically targeted immunoglobulin gene transcripts. These data sets are created using massively multiplexed PCR reactions that are subsequently sequenced on the Illumina platform. Multiplex amplification strategies have been used to explore the T‐cell^10^, ^11^ and B‐cell repertoires,^12^, ^13^, ^14^, ^15^ and biases, omissions.^10^, ^11^, ^16^, ^17^, ^18^and PCR artifacts have been detected in the data sets.^10^

We started this project with the hypothesis that our unamplified data set would provide comparable results to those seen in the commercially amplified data sets. However, we are unaware of any immunoglobulin repertoire studies that have been done to compare data obtained using amplification techniques compared to the repertoire in a total RNASeq library. Concurrent with performing these analyses, we discovered that the required sample preparation for commercial sequencing also varied. Therefore, we found it necessary to examine the impact of sample preparation as part of our effort. This manuscript describes our results comparing a data set generated using unamplified total RNA (TRNA) to commercially amplified data sets. We examined the role of commercial amplification and cDNA generation methods as well as the impact of the starting material on sequence output.

## 2 MATERIALS AND METHODS

### 2.1 KSU RNA preparation

RNA was prepared as outlined in Rettig et al.^9^ Briefly, RNA was extracted from the spleens of four 9‐week‐old female C57Bl/6 J mice. TRNA was submitted to the Kansas State University Integrated Genomics Facility for sequencing and cDNA was prepared using standard Illumina protocols. cDNA made from the TRNA using random hexamer primers and oligo‐dT selection, was size selected to 275‐800 bp length and sequenced on the Illumina MiSeq at 2 × 300 bp using Illumina's protocol. No additional amplification beyond that required by Illumina preparation was used and we consider these samples nonspecifically amplified, or “unamplified.”

### 2.2 Commercial sample preparations

mRNA was extracted from the TRNA isolated from the sample used for the KSU data set using the PolyATtract mRNA isolation system (Promega, Fitchburg, WI) following manufacturer's instructions. RT‐PCR for samples amplified with Avian Myeloblastosis Virus (AMV) reverse transcriptase–based sample preparation was performed using the Access RT‐PCR System (Promega, Fitchburg, WI) following the manufacturer's instructions. RT‐PCR for Moloney Murine Leukemia Virus (MMLV) reverse transcriptase–based sample preparation was performed using the SuperScript III First‐Strand Synthesis System (Invitrogen, Carlsbad, CA) following manufacturer's instructions. Starting material for RT‐PCR was either TRNA from the KSU data set or the purified mRNA. RT‐PCR products were submitted to Adaptive Biotechnologies (Seattle, WA) **(**Com1) on dry ice following company protocols. TRNA and mRNA (unamplified) were submitted to iRepertoire, Inc (Huntsville, AL) (Com2) on dry ice following company protocols.

### 2.3 Bioinformatic analysis

KSU sequencing results were analyzed as described previously.^9^ Briefly, sequencing results were quality controlled. Antibody‐specific sequences were isolated and submitted to ImMunoGeneTics (IMGT)^19^ for analysis. Individual sequences were assigned a unique ID by the sequencing machinery during Illumina sequencing and were used to identify unique sequences. The sequence containing the most high‐quality information was retained for further analysis as outlined in Rettig et al.^9^ No further filtering of reads was performed. Both commercial sequencers provided their own bioinformatic analyses of the sequencing results. The raw sequencing results from Com1 and Com2 were also submitted to IMGT for analysis and subjected to the standard KSU bioinformatics pipeline. IMGT's nomenclature and classifications were used throughout this paper. We assessed all functional V‐gene segments as identified by IMGT. We also include three putative functional genes (V5S21, V1S100, and V3S7) which were detected in rearranged transcripts (containing a CDR3 C‐xx‐W motif or class switched) in our previous analysis of the normal C57BL/6 repertoire.^20^ IMGT's High‐V Quest occasionally assigned multiple potential V‐gene segments to a single sequence, likely due to incomplete capture of the entire V‐gene sequence or high homology between gene segments.

In all IMGT processed data, sequences that contained two possible V‐gene segment possibilities were assigned a weighted value of 0.5 per sequence, as opposed to one for full matches. Sequences with V‐gene segments that were assigned more than two potential matches were excluded from analysis. Initial results were tabulated using the companys’ proprietary bioinformatic results. However, to determine the role of bioinformatic handling of the data, some of Com1 and Com2 data were subjected to the standard KSU bioinformatic workflow analysis and CDR3 analyses.^9^

### 2.4 Statistical tests

All statistical analyses were carried out using GraphPad Prism (Version 6.0). Paired T tests were performed using the raw read counts. Coefficient of determinations (R^2^) were performed by comparing the percent of repertoire between animals. Percent of repertoire is determined by dividing the read count for a specific V‐gene segment by the total number of reads detected and multiplying by 100.

## 3 RESULTS

Most studies examining immunoglobulin repertoires use amplification to increase the depth of sequencing, but amplification comes with some drawbacks. We wanted to assess the comparability of amplified and non‐amplified data from identical samples. In preparation to do this comparison, we found that different commercial amplification methodologies required different types of sample preparation. For example, sample submission for the Com1 data sets required a cDNA sample. The Com1 process amplified the resulting cDNA using proprietary primers and sequencing on the Illumina platform. After an initial submission showed a low correlation between the Com1 sequencing and the KSU data set (data not shown), we hypothesized that cDNA preparation plays a role in determining the amplified repertoire. To test this hypothesis, we assessed the role of starting material (mRNA or TRNA), reverse transcriptase (AMV vs MMLV), and primer templates (oligo‐dT or random hexamer) on the sequenced B‐cell immunoglobulin repertoire. Com2 submissions required the submission of TRNA, rather than cDNA.

### 3.1 Assessment of transcriptional read counts

Com1 amplified data sets returned between 7084 and 1 263 003 sequences, dependent on the preparation method. mRNA starting material yielded more total transcriptional reads than TRNA (*P* = 0.013, two tailed matched T test; Table 1). Generally, the AMV reverse transcriptase and random hexamer primers tended to yield higher numbers of transcripts. The use of AMV and random hexamer primers resulted in more total productive reads in three out of four of the comparisons directly comparing primers, however, the overall differences were not statistically different (*P* > 0.05, two‐tailed matched T test; Table 1). In the Com2 data set, we found a moderate number of reads, about one‐half of those detected in the highest Com1 numbers. These compare to 11 200 sequence reads containing a CDR3 generated in the KSU data set using a total MiSeq approach.

**Table 1:** Total number of productive reads per data set

	KSU^a^	Com1^a^								Com2^a^	
		mRNA^b^				TRNA^b^				mRNA^b^	TRNA^b^
		AMV^c^		MMLV^c^		AMV^c^		MMLV^c^			
		dT^d^	Hex^d^	dT^d^	Hex^d^	dT^d^	Hex^d^	dT^d^	Hex^d^		
Total Productive Reads	11 200^e^	553 521	1 263 003	883 532	1 035 461	7 975	6 867	208 979	220 772	637 214	766 075

a Sequencing technique (Com1 and Com2 are amplified data sets).

b Starting material (mRNA, messenger RNA; TRNA, total RNA).

c Reverse transcriptase (AMV, Avian Myeloblastosis Virus; MMLV, Moloney Murine Leukemia Virus).

d Primer (dt, Oligo dT; Hex, random hexamer).

e An additional 27 896 reads were used for V‐gene segment usage assessment. These sequences were not long enough for CDR3 detection.

### 3.2 Determination of sequencing reproducibility

To assess the repeatability of the amplified Com1 and Com2 data sets, we examined the correlation of V‐gene segment usage. In the C57BL/6 mouse, the V‐gene segment is the most varied in the heavy chain (IgH locus) comprising a total of 109 functional V‐gene segments, three putative function V‐gene segments, and alleles compared to 19 for the D‐gene segment and four for the J‐gene.^21^, ^22^ The nucleotide sequences in the V‐gene segments are also highly varied and require a complex multiplex PCR to amplify. Correlations were assessed using the data provided by the commercial vendor's proprietary bioinformatics.

**Figure 1:**
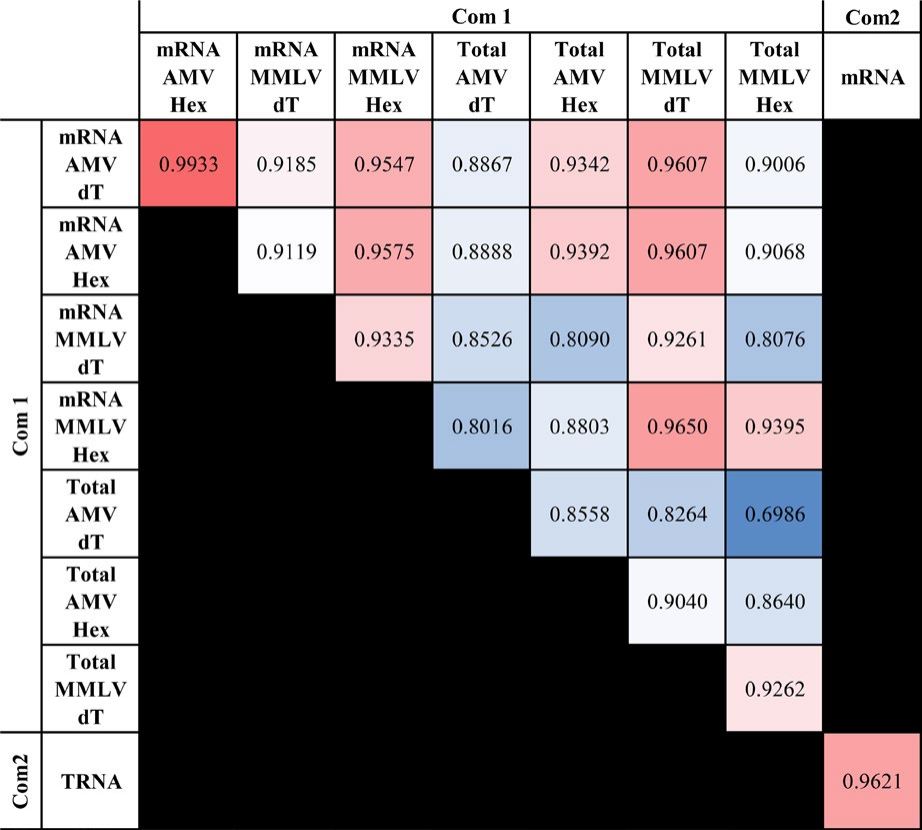
R2 values of sequencing technical replicates. The percent of repertoire for each V‐gene segment detected was compared between technical replicates. The highest R2 are dark read, while the lowest R2 are blue

Non‐strain specific V‐gene segment assignments accounted for between 0.84% and 1.46% of the sequencing results from Com1 and 1.74% and 1.41% for Com2 (Table 2). Although there were differences in the immunoglobulin gene transcripts detected, there was a high correlation in the V‐gene sequences detected among the different technical replicates in the Com1 data (R^2^ range from 0.6986 to 0.9933, all *P* < 0.0001) (Figure 1). The R^2^ between technical replicates in Com2 was 0.9621 (*P* < 0.0001). We also examined the reproducibility of two technical replicates of KSU sequencing used for a different analysis and obtained an R^2^ of 0.9996 for Mouse 32, and 0.9995 for Mouse 39 (*P* < 0.001) showing high levels of reproducibility between KSU sequencing runs. Therefore, although total transcripts generated varied with sample preparation, the V‐gene segments that were detected were consistently detected using two different commercial approaches.

**Table 2:** Percent of non‐C57BL/6 V‐gene segments detected per data set

	Com1^a^								Com2^a^	
	mRNA^b^				TRNA^b^				mRNA^b^	TRNA^b^
	AMV^c^		MMLV^c^		AMV^c^		MMLV^c^			
	dT^d^	Hex^d^	dT^d^	Hex^d^	dT^d^	Hex^d^	dT^d^	Hex^d^		
% Non‐B6 V‐Gene segments	0.92	0.88	0.84	1.17	1.30	1.46	1.02	1.14	1.74	1.41

a Sequencing technique (Com1 and Com2 are amplified data sets).

b Starting material (mRNA, messenger RNA; TRNA, total RNA).

c Reverse transcriptase (AMV, Avian Myeloblastosis Virus; MMLV, Moloney Murine Leukemia Virus).

d Primer (dt, Oligo dT; Hex, random hexamer).

### 3.3 Impact of amplification on V‐gene segment detection

**Figure 2:**
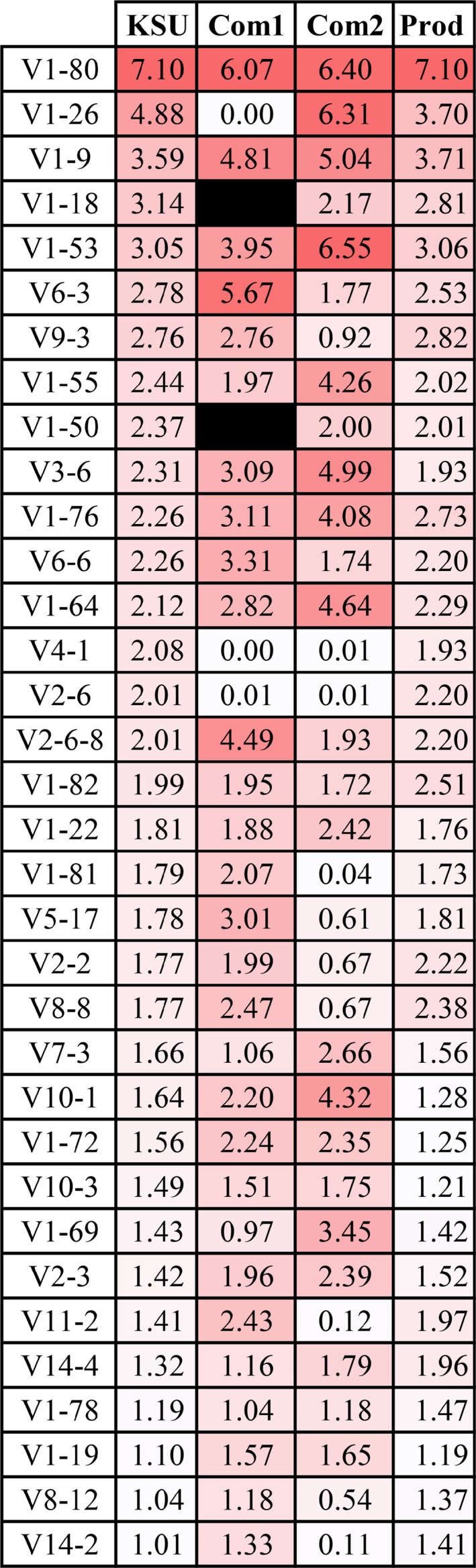
Percent of repertoire for high‐frequency V‐gene segments among data sets. Percent of repertoire for the KSU, Com1 (mRNA‐MMLV‐hex), Com2 (mRNA), and Prod (productive only sequences from the KSU data set) are displayed. The highest value percent of repertoire is dark read while the lowest are white. Black boxes represent no detected reads (true zero). Rounded zeros are represented as 0.0

The unamplified KSU approach produced a data set where a total of 112 V‐gene segments were detected while the Com1 data sets contained between 85 and 100 V‐gene segments. The Com2 mRNA data set contained 99 detectable V‐gene segments and the TRNA contained 100. Comparisons of V‐gene segments in the Com1 data set to the KSU data set showed moderate R^2^ values (0.4457 to 0.5841, all *P* < 0.0001) (Table 3). The Com2 data sets also showed moderate R^2^ of 0.6695 for mRNA and 0.6607 for TRNA (all *P* < 0.0001). To determine why there were differences in V‐gene detection, we compared the results from the most commonly detected V‐gene segments in the KSU data sets to their frequencies in the Com1 and Com2 data sets. The protocol for Illumina sequencing uses mRNA selection, SuperScriptIII reverse transcriptase, and random hexamer primers. To best compare results, we used the Com1 mRNA‐MMLV‐Hex data set and the Com2 mRNA data set using the top 34 V‐gene segments in the KSU data set. These V‐gene segments comprise over one percent of the detected repertoire and are considered highly expressed. In the Com1 data set, of these 36 highly expressed V‐gene segments, five (V1‐26, V1‐18, V1‐50, V4‐1, and V2‐6) were detected at twofold lower frequency than in the KSU data set (Figure 2). These five V‐gene segments were either absent or were near zero percent of the repertoire (Figure 2). Of these same top 34 V‐gene segments in the Com1 data set, two (V6‐3 and V2‐6‐8) were detected at twofold greater than the KSU data set (Figure 2). The R^2^ for these top 36 V‐gene segments to the KSU data set was 0.1783 (*P* = 0.0128).

**Table 3:** Correlations of data sets to unamplified KSU data set and read counts

	Com1^a^								Com2^a^	
	mRNA^b^				TRNA^b^				mRNA^b^	TRNA^b^
	AMV^c^		MMLV^c^		AMV^c^		MMLV^c^			
	dT^d^	Hex^d^	dT^d^	Hex^d^	dT^d^	Hex^d^	dT^d^	Hex^d^		
R^2^ to KSU Dataset	0.5677	0.5773	0.4496	0.5517	0.4457	0.5606	0.5554	0.5841	0.6695	0.6607
Assessed V‐Gene Segments	506 503	151 104	1 749 618	1 245 999	267 946	5666	267 946	302 057	626 093	755 280

a Sequencing technique (Com1 and Com2 are amplified data sets).

b Starting material (mRNA, messenger RNA; TRNA, total RNA).

c Reverse transcriptase (AMV, Avian Myeloblastosis Virus; MMLV, Moloney Murine Leukemia Virus).

d Primer (dt, Oligo dT; Hex, random hexamer).

We also compared the Com2 mRNA data set to the same 34 V‐gene segments from the unamplified KSU data. Of the 34 V‐gene sequences, nine (V9‐3, V4‐1, V2‐6, V1‐81, V5‐17, V2‐2, V8‐8, V11‐2, V14‐2) were detected at a twofold or lower level than in the KSU data set (Figure 2). Five other V‐gene segments (V1‐53, V3‐6, V1‐64, V10‐1, and V1‐69) were detected at twofold higher levels than those found in the KSU data set (Figure 2). The correlation of these top 34 V‐gene segments was better than the Com1 R^2^ at 0.4098 (*P* <0.0001).

Our normal workflow methods include the use of functionally productive and unknown transcripts for analysis^9^. This inclusion helps balance the lower read numbers obtained with unamplified sequences. We performed the same analysis as above between our productive + unknown data set used above, with our productive only data set. We detected a total of 104 V‐gene segments. Those not detected in the productive only list (V3S7, V6‐7, V6‐4, V1‐62‐1, V5‐12‐4, V1‐17‐1, and V6‐5) comprised less than 0.7% of the repertoire. The correlation coefficient was high at 0.9596 (*P* < 0.0001), and there were no changes at greater than twofold of the productive + unknown data set (Figure 2). These analyses reveal that the addition of unknown functionality V‐gene segments does not significantly alter the repertoire.

### 3.4 Direct comparisons of amplified and unamplified data sets

The comparisons in V‐gene use were made using the bioinformatics provided by the commercial ventures. To standardize the data handling to remove bioinformatic reasons for the differences in data, we processed the sequencing results from the Com1 mRNA‐MMLV‐Hex and Com2 mRNA data sets using the KSU bioinformatics work flow.^9^

The KSU bioinformatic treatment of the Com1 data set correlated moderately with the commercially provided bioinformatics (R^2^ = 0.4795, *P* < 0.0001). After processing the Com1 data with the KSU bioinformatics pipeline, the R^2^ to the KSU data set increased slightly from 0.5517 (Table 3) with the original bioinformatics to 0.5649 (*P* < 0.0001) with the adjusted bioinformatics. However, nine V‐gene segments were detected in the Com1 data set using the KSU bioinformatics workflow that were not originally detected using the commercially provided bioinformatics (Supporting information Figure S1). When we processed the Com2 data using the KSU bioinformatic pipeline, the Com2 data set was highly correlated with the original commercially provided bioinformatics treatment (R^2^ = 0.9860, *P* < 0.0001). When we compared Com2 data set processed with the KSU bioinformatics pipeline to the KSU RNASeq data set, the data still only had an R^2^ = 0.6791 (*P* < 0.0001). The KSU bioinformatics workflow detected an additional four V‐gene segments that were not detected by the commercial bioinformatics (Supporting information Figure S1).

When we reanalyzed the bioinformatics data from Com1 and Com2 using the KSU pipeline, we detected gene segments that were not detected in the original commercially provided bioinformatics. However, the inclusion of these gene segments, did not greatly improve the R^2^ between the amplified data sets and the KSU RNASeq data. In the Com1 data set, some gene segments (V1‐26, V1‐18, V1‐50, V2‐9‐1) were not detected or only detected at low levels in the original bioinformatics but were detected at high levels (>1%) in the KSU/IMGT processed data (Supporting information Figure S1). The three additional V‐gene segments detected in the Com2 data set (V2‐5, V1‐62‐2, and V1‐62‐3 were found in less than <0.3% of the repertoire (Supporting information Figure S1). These changes were not sufficient to significantly improve R^2^ values.

### 3.5 Impact of amplification on the reproducibility of CDR3 detection

The absence of some V‐gene segments in the Com1 and Com2 data compared to the KSU data was a concern. It precludes a complete picture of the V‐gene repertoire. Nevertheless, amplified sequencing of the antibody repertoire is thought to provide an advantage in that the depth of coverage is increased over unamplified data sets due to the number of reads generated. To determine how extensive the discrepancy is between amplified and unamplified data, we assessed the read depth (number of reads generated) and resampling efficiency of CDR3 (number of unique CDR3s resampled between replicates) using technical replicates of samples sequenced with the various sequencing techniques. As anticipated, amplified data sets had both higher total read numbers and unique CDR3 numbers (Table 4).

**Table 4:** Unique CDR3 sequences in the KSU, Com1, and Com2 data sets

	KSU^a^	mRNA‐MMLV‐Hex (Com1)^a^	mRNA^a^ (Com2)
Read Count^b^	11 200	1 035 461	637 214
Unique CDR3 Sequences^c^	6668	180 266	146 231

a Sequencing data set.

b Total number of reads obtained per data set.

c Total number of unique CDR3 AA sequences.

**Figure 3:**
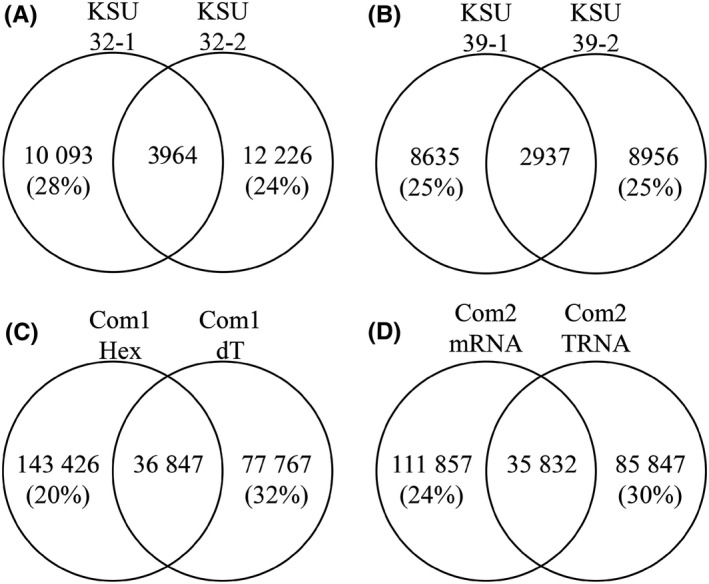
Overlap of CDR3 sequence detection between technical replicates. CDR3 amino acid sequences were compared between technical replicates. Sequences unique to one data set are displayed in the outer circles. Sequences shared between data sets are in the overlap. Percent of shared CDR3 sequences is displayed in parentheses in the outer circles. (A) KSU data sets 32‐1 and 32‐2. (B) KSU data sets 39‐1 and 39‐2. (C) Com1 data sets mRNA‐MMLV‐Hex and mRNA‐MMLV‐dT. (D) Com2 data sets mRNA and TRNA

Resampling/reproducibility has been assumed to improve with the depth of coverage. We had the unique opportunity to compare sequencing data from the same biological material subjected to multiple sequencing methodologies. We have also had the opportunity to do technical replicates on multiple samples subjected to RNASeq or amplification procedures. This allowed us the ability to look at CDR3 sampling reproducibility and to determine if amplification provided any advantage in CDR3 reproducibility. For the KSU unamplified data set, two C57BL/6 J mouse spleen RNA samples (#32 and #39) were sequenced independently two times each and the CDR3s sampled were compared. In the KSU data set, 32‐1 shared 28% of its total unique reads with 32‐2. (Figure 3). 32‐2 shared 24% of its reads with 32‐1 (Figure 3). KSU data set, 39, showed 25% overlap of their total unique reads between each sampling (Figure 3). For the Com2 data, since there was such a strong correlation between the sequence output between mRNA and TRNA samples of C57BL/6 J spleen samples (R^2^ = 0.9644), we considered these technical replicates. The mRNA data set shared 24% of its sequences with the TRNA data set while the TRNA data set shared 30% of its sequences with the mRNA data set. We also examined the resampling efficiency in the Com1 data set using the spleen mRNA‐MMLV data sets that were reversed transcribed with two different primers, random hexamer and oligo‐dT. Although this was not a perfect technical replicate, there was an R^2^ of almost 0.94 in V‐gene segments detected (Figure 1). Therefore, we felt these served as “incipient” technical replicates. The random hexamer data set shared 20% of the detected CDR3s and the oligo dT data set shared 32% of its CDR3 sequences. Therefore, regardless of data set, there was an average of 26%‐27% sampling overlap regardless of whether amplification was performed or not.

**Figure 4:**
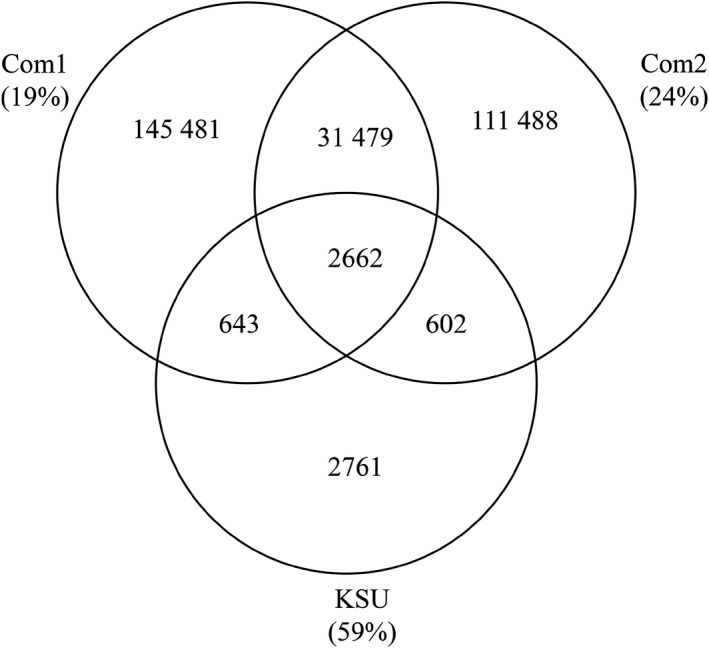
CDR3 sequence capture among Com1, Com2, and KSU data sets. CDR3 amino acid sequences were compared among the Com1 mRNA‐MMLV‐Hex, Com2 mRNA, and the KSU data sets. Percent of the repertoire shared with at least one other data set is listed in parentheses

We also assessed the overlap in the detected CDR3s between the Com1 data set (mRNA‐MMLV‐Hex), Com2 data set (mRNA), and the KSU original data set to determine the extent of the overlap of CD3 detection using the different methods. From the 295 116 CDR3 unique sequences that were detected, 2662 of those sequences were shared among all three data sets (Figure 4). The amplified data sets from Com1 and Com2 shared the most CDR3 sequences between them with 34 141 sequences found in both data sets (Figure 4). The KSU data set shared 59% of its detected CDR3 sequences with the Com1 and Com2 data sets (Figure 4). These data are consistent with the lower depth of sequencing of the unamplified data set compared to the Com1 and Com2 data sets where 19%‐32% overlap occurred in detected CDR3 sequences.

### 3.6 Detection of high‐frequency CDR3s

**Figure 5:**
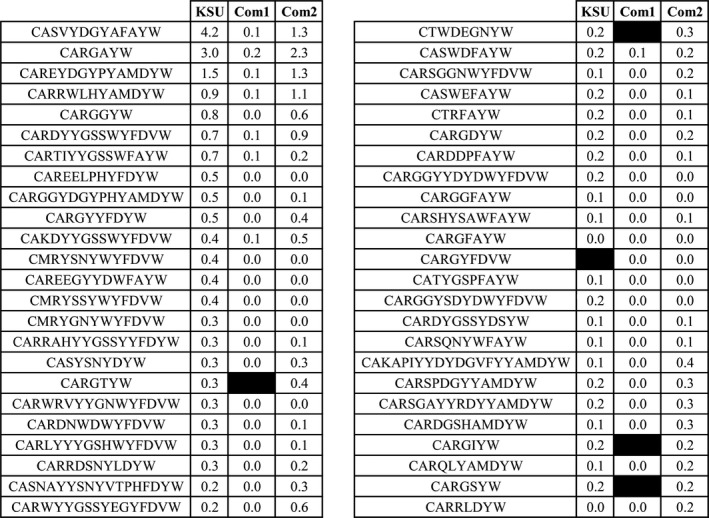
High‐frequency CDR3s detected among the Com1, Com2, and KSU data sets. The top 25 CDR3s from each data set (48 total) were compiled and percent of repertoire compared. Black boxes represent no detected reads (true zero). Rounded zeros are represented as 0.0

To gauge whether the highest frequency CDR3s can be detected by the different techniques, we assessed the 25 highest frequency CDR3s from each sequencing method. This resulted in a total of 48 unique CDR3s from the three different methods (Figure 5). The KSU data set detected all but one (CARGYFDVW) of these 48 sequences, the Com1 data set failed to detect four sequences (CARGTYW, CTWDEGNYW, CARGIYW, and CARGSYW) and the Com2 data set detected all 48 sequences (Figure 5). The CDR3s that were not detected in the Com1 data set, did use V‐gene segments that were detected in the data set. These data show that although the depth of sequencing of the KSU data set was about 10% of the amplified data sets, the data set still captured 98% of the highest frequency CDR3’s.

### 3.7 CDR3 frequency assessment

While the high‐frequency CDR3s were shared among at least two sequencing runs, most of the CDR3 sequences detected were unique to each sequencing run (Figure 4). To determine the frequency of unique CDR3 sequences, we compared the most frequent, least frequent, and average percent of the measured CD3 repertoire (Table 5). The highest frequency CDR3 was detected at 4.16%, 0.22%, and 2.26%, and of the repertoire for the KSU, Com1, and Com2 data sets, respectively (Table 5). The lowest frequency CDR3s, representing only a single detected read, were 0.0088%, 0.0004%, and 0.0002%, of the repertoire for the KSU, Com1, and Com2 data sets, respectively (Table 5). The average detection level for the KSU data set was 0.015%, 0.0006% for the Com1 data set, and 0.0007% for the Com2 data set (Table 5).

**Table 5:** CDR3 AA sequences frequencies in the whole and unique repertoire

	Whole repertoire^a^			Unique repertoire^a^		
	KSU	Com1	Com2	KSU	Com1	Com2
Minimum^b^	0.008758	0.000446	0.000221	0.008758	0.000446	0.000221
Maximum^b^	4.165500	0.216969	2.259680	0.035032	0.005804	0.026044
Average^b^	0.014997	0.000555	0.00684	0.008929	0.000478	0.00290

a Repertoire sampled (Whole repertoire – includes all sequences, unique repertoire – sequences unique to a single analyzed repertoire).

b Minimum or maximum frequency in the repertoire.

We also examined the frequency of CDR3s that were unique to each data set. Overall, the maximum and the average frequencies of the data sets were reduced compared to the whole repertoire (Table 5). This demonstrates that the unique reads in each data set were most likely transcripts from low frequency B cells. Moreover, these data suggest that even without amplification, the KSU data set was detecting the most prevalent CDR3s and many low‐frequency sequences.

## 4 DISCUSSION

Illumina sequencing of total RNA from mouse spleen is able to capture a representative sample of the splenic B‐cell repertoire. This snapshot of the repertoire, while producing less reads than amplified data sets, detected more V‐gene segments than data sets that used two different amplification strategies and captures 98% of the high frequency CDR3s found in the amplified data sets. While amplified data sets provide more CDR3 depth of coverage, the unamplified data sets produced from an RNASeq allow for further data mining, eliminate as much primer bias as possible and provides an accurate representation of the repertoire.

Sequencing requirements of the B‐cell receptor are more challenging than those of the T‐cell receptor. There are no consensus sequences to reference.^10^ Additionally, transcripts from the germline which are not successfully rearranged can be detected.^23^ Therefore, avoiding bias is one of the main priorities for antibody repertoire sequencing.^16^ PCR errors are accumulated through the amplification process which can falsely inflate the repertoire or they can add suspected mutations that do not exist^10^, ^17^, ^24^, ^25^ and they may not be distinguishable from low level mutations that actually do exist in the repertoire.^24^, ^26^ PCR biases can be introduced because of primer binding properties, CG content, mispriming, nonspecific binding, and errors during replication.^16^, ^27^, ^28^, ^29^ A specific issue in targeting antibody gene segments is primer annealing efficiencies since the gene segments that make up the murine IgH locus are similar, though not identical.^28^ The biases inherent in the multiplex PCR can lead to false repertoire skewing, gene frequency inaccuracies, and a less comprehensive view of the repertoire.^25^, ^30^, ^31^ The development of these multiplex primers, is highly challenging.^32^ Work by Bashford‐Rogers^33^ shows that there is little difference between RNA‐capture, 5’RACE or PCR amplification in humans but others^27^ have shown that PCR does create biases and 5’ RACE helps reduce these.

Our results demonstrate some of the issues of assessing B‐cell repertoires using massively multiplexed PCR reactions. While reproducibility for technical replicates was moderate to high, there was a large range of total read numbers across methodologies. Given the increase in read results with mRNA samples compared to TRNA samples, the authors hypothesize that mRNA increases the availability of antibody specific transcripts, increasing the overall read counts. Additionally, the reverse transcriptase plays a role in sequence generation. AMV reverse transcriptase has an RNase H activity, where MMLV reverse transcriptase does not.^34^ This activity may cleave RNA transcripts prior to completion of amplification, resulting in shorter reads with inadequate length for amplification. The use of oligo‐dT primers will begin reverse transcription from the most 3’ end of the transcript. The target of amplification is around 1.5 kilobases upstream of the poly‐A tail,^35^ likely leading to shorter reads and failure to amplify the region of interest. Random hexamer primers may be able to overcome this bias due to their random priming nature.

In addition to variations in read depth, the correlations of these data sets to the unamplified KSU data sets were low to moderate, even when the same bioinformatics processing was employed. Of significant concern is the Com1 and Com2 sets failed to detect 13 V‐gene segments that were detected by the unamplified KSU RNASeq data set. The absence of these genes draws into question how one compares the various data sets with correlation coefficients that are below 0.7 when technical replicates of the same sequencing are greater than 0.99. Although Carlson et al argues that amplification methodologies can capture the entire repertoire, there are concerns.^11^ Even when we only looked at the V‐gene families detected at the highest frequency there were omissions. Of the 34 V‐genes that we categorized as “high frequency” (>1% of the repertoire), Com1 found lower detection levels (defined as less than twofold that found in the KSU data set) for five gene segments (V1‐26, V1‐18, V1‐50, V4‐1, and V2‐6) and nine (V9‐3, V4‐1, V2‐6, V1‐81, V5‐17, V2‐2, V8‐8, V11‐2, V14‐2) for the Com2 data set. These results suggest that those methods are skewing the reported repertoire by missing or underreporting those high frequency V‐gene segments. Interestingly, two V‐gene segments (V4‐1 and V2‐6) were underrepresented in both data sets, but the other V‐gene segments were unique.

The failure of primers to capture specific V‐genes is not a new discovery, as primers failed to adequately sequence hybridomas previously.^18^, ^36^ “Universal” primers for the human antibody repertoire do exist, but some questions remain if they cover the entire repertoire.^37^ The difficulty in developing a “universal” or even highly comprehensive primer set for the mouse is likely due to their highly varied leader sequences, V‐gene segments, and framework regions. Primer design would have to rely on massively multiplexed reactions and/or degenerate primers. Additionally, the most commercially viable amplification methods would need to amplify across multiple common strains adding additional levels of complexity. Indeed, in our attempts to design “universal” primers, we found a minimum of 11 primer sets would be needed to detect V‐genes associated for each isotype. Even then, there were still issues with matching PCR conditions and efficiency. Methods to overcome the biases detected in amplification have been developed, such as the use of 5’ RACE^26^, ^38^, ^39^ and using molecular barcodes or identifiers.^40^, ^41^ However, these methods are expensive and have their own draw backs. Replication of the entire repertoire using 5’RACE would still require the use of multiple constant region primers, leading to the same multiplexing issues. Barcoding can have errors and chimeric reads making repertoires difficult to reconstruct.^42^ This latter issue is not a problem with our RNASeq data.

While bias exists in the massively multiplexed amplification process, there may be some sequencing errors in the unamplified KSU data set as well. While not specifically amplified for antibody sequences, random hexamers and oligo‐dT capture beads are used prior to sequencing to generate the library^43^ and some biases have been observed in random hexamer binding.^44^ The use of oligo‐dTs can result in enhancement of the 3’ end of transcripts.^45^ We do not think this is particularly problematic since Illumina sequence methodology aims to reduce bias in their library preparations by combining the random hexamer and oligo‐dT capture. Additionally, while all libraries were sequenced on the Illumina platform, 3.19% of high‐quality Illumina reads contain false base calls, which are impossible to differentiate using normal bioinformatics methods.^10^ Overrepresentation of specific dinucleotides can also be detected in sequencing which are not related to primer usage.^46^ Therefore, although we hope to reduce bias and omission by doing RNASeq, we still have some technical issues that keep the data set from being a perfect reflection of the repertoire. Multiple technical replications help reduce the impact of this problem.

One analysis that was not pursued in this current investigation was the identification of clonally related sequences. We were specifically interested in the functional antibody repertoire present in the spleen and focused our analysis at the transcript level. We acknowledge that the overrepresentation of some sequences may be likely within our data sets since we do not use barcoding or clonality analysis to collapse similar mRNA sequences. Nevertheless, the overrepresentation of a specific sequence by an overly productive cell is also representative of cellular activation of transcription, and likely, functional antibody protein in the body.^47^, ^48^

The lack of amplification also results in varying sequence lengths in our data set. We selected 40 nt as our minimum cutoff to provide us enough information to detect V‐, D‐, and J‐ gene segments. While some short sequences were included in the data analysis, our overall average sequence length was 287 nt, with productive sequences averaging 331 nt and unknown sequences averaging 270 nt (data not shown). Overall, less than 0.5% of the sequences analyzed where less than 100 nt long (data not shown). Therefore, we do not think that sequence length is an issue in this study.

Although there are issues using massively multiplexed PCR reactions, there are advantages that may overshadow the disadvantages. For example, increased sequencing depth (eg, 1 260 000 reads vs 11 200 complete reads), better low‐frequency CDR3 detection (20‐fold more unique CDR3 sequence than the unamplified KSU data set), and sequencing costs can be lower than an Illumina MiSeq run (by integrating multiplexing/barcoding).

Although amplification provides more detail in the CDR3 repertoire, if one is interested in the B‐cell clones that are most prevalent, then RNASeq does not appear to be at a disadvantage. CDR3 resampling was similar (20%‐32%) regardless of method. Additionally, when examining high frequency CDR3s, the unamplified data set only failed to detect a single unique CDR3 sequence, while the Com1 data set failed to detect four. Without using an amplification method with unique barcoding, such as that used in Shugay et al^49^ it is impossible to tell which unique CDR3s are, in fact, correctly identified new sequences and which involve miscalls, leading to false diversity. As our current paper does not focus on the actual diversity of the repertoire, and instead focuses on the differences among sample preparations and sequencing methods, we do not attempt to identify false call unique CDR3s as doing so may falsely reduce diversity. Instead, we used strict read quality cutoffs to prevent low quality base calls from being included in analysis, and we point to our highly overlapping V‐gene segment usage (R^2^ = 0.9995 among multiple sequencing replicates to assure us that we minimized artifacts. Additionally, all sequencing was performed on the Illumina platform, so false base call rates should be similar across methodologies which we were focused on in this study.

When preparing for antibody repertoire sequencing, multiple factors must be considered within the framework of the specific biological questions being asked. This includes needed coverage, cost, and starting material.^39^ Additionally, it is important to consider that all repertoire sequencing is merely a snapshot of a constantly shifting image.^25^ We will also never be able to fully capture the full diversity of the B‐cell immunoglobulin repertoire, which is estimated to range from 10^6^‐10^7^ possible unique rearrangements and mutations^25^, ^39^ to as much as 10^13^.^15^ The failure of the KSU data set to detect rare clones compared to the amplified data sets is likely due to this; but even the amplified data sets only sampled a fraction of the total repertoire. Therefore, one must decide how “deep” is adequate for the question being addressed.

During this investigation, we also had to address the issue that starting material may influence the quality of one's sequencing. mRNA as a starting template increased reads up to two orders of magnitude. Furthermore, random hexamer primers and MMLV reverse transcriptase generally yielded higher read count results. Interestingly, the use of mRNA, with MMLV reverse transcriptase and hexanucleotide primers is most technically like that used in Illumina sequencing. However, additional data will be needed to confirm our observations since we did not pursue this aspect of the study in detail and the replicate number did not allow for robust statistical comparisons.

In conclusion, we have demonstrated that sequencing of unamplified splenic RNA provides a realistic snapshot of the total splenic B‐cell repertoire. We also have demonstrated that a good understanding of the bioinformatics work flow and reporting of the methodology is critical and cannot be understated. We understand that there are cellular biases and transcript stability differences within B‐cell subpopulations.^50^, ^51^ However, for the purpose of assessing a whole tissue B‐cell repertoire, unamplified RNASeq can provide a glimpse of the most prevalent B‐cell clones. The unamplified approach could just as well be applied to specific cell populations when the application requires it. Moreover, an unamplified data set may detect V‐gene segments that amplified data sets miss.

## CONFLICTS OF INTEREST

The authors declare no conflict of interest.

## AUTHOR’S CONTRIBUTION

T.A.R. and S.K.C. contributed to the experimental design, data analysis, and manuscript preparation and editing. M.J.P. contributed to experimental design and manuscript editing.
