# Supplementary material for: A comparison of unamplified and massively multiplexed PCR amplification for murine antibody repertoire sequencing
Source: FASEB Bioadv. 2018 Oct 25;1(1):6–17. doi: 10.1096/fba.1017 (PMC6996338; doi:10.1096/fba.1017)
Supplement: Supplementary file 1 [file FBA2-1-6-s001.pdf]

Supplemental Figure 1

	KSU	Com1 (Orig)	Com1 (IMGT)	Com2 (Orig)	Com2 (IMGT)
V1-80	7.10	6.07	2.89	6.40	6.45
V1-26	4.88	0.00	2.95	6.31	6.17
V1-9	3.59	4.81	3.23	5.04	5.07
V1-18	3.14		2.85	2.17	2.37
V1-53	3.05	3.95	4.37	6.55	6.59
V6-3	2.78	5.67	3.33	1.77	1.83
V9-3	2.76	2.76	2.58	0.92	0.92
V1-55	2.44	1.97	2.31	4.26	4.30
V1-50	2.37		1.14	2.00	2.02
V3-6	2.31	3.09	2.72	4.99	5.03
V1-76	2.26	3.11	1.22	4.08	4.12
V6-6	2.26	3.31	1.86	1.74	1.71
V1-64	2.12	2.82	3.51	4.64	4.69
V4-1	2.08	0.00	0.63	0.01	0.01
V2-6	2.01	0.01	0.17	0.01	0.96
V2-6-8	2.01	4.49	0.17	1.93	0.96
V1-82	1.99	1.95	3.01	1.72	1.73
V1-22	1.81	1.88	2.28	2.42	2.44
V1-81	1.79	2.07	2.29	0.04	0.04
V5-17	1.78	3.01	2.89	0.61	0.61
V2-2	1.77	1.99	2.26	0.67	0.69
V8-8	1.77	2.47	1.03	0.67	0.67
V7-3	1.66	1.06	0.79	2.66	1.81
V10-1	1.64	2.20	1.89	4.32	4.39
V1-72	1.56	2.24	3.08	2.35	2.08
V10-3	1.49	1.51	1.14	1.75	1.58
V1-69	1.43	0.97	1.03	3.45	3.23
V2-3	1.42	1.96	1.32	2.39	2.41
V11-2	1.41	2.43	0.12	0.12	0.12
V14-4	1.32	1.16	1.91	1.79	1.80
V1-78	1.19	1.04	0.66	1.18	1.21
V1-19	1.10	1.57	1.72	1.65	1.73
V8-12	1.04	1.18	0.67	0.54	0.55
V14-2	1.01	1.33	1.87	0.11	0.11
V1-52	0.99		0.40	0.02	0.02
V7-1	0.98	1.82	0.24	2.16	1.74
V1-15	0.98	0.73	1.36	0.04	0.04
V5-4	0.92	1.13	1.31	1.25	1.26
V1-7	0.89	0.65	0.90	1.11	1.06
V1-39	0.88	1.65	1.98	0.35	0.35
V5-16	0.84	0.85	0.54	0.42	0.43
V2-9	0.83	0.68	0.87	0.85	0.68
V9-1	0.80	0.87	0.61	0.45	0.45
V2-9-1	0.79	0.00	1.16	0.00	0.22
V1-75	0.73	1.30	1.36	1.06	1.07
V1-4	0.69	0.39	0.41	0.99	1.16
V2-5	0.68		0.64		0.28

	KSU	Com1 (Orig)	Com1 (IMGT)	Com2 (Orig)	Com2 (IMGT)
V3-8	0.68	0.70	0.52	0.14	0.14
V1-12	0.63	0.65	0.41	0.40	0.37
V1-74	0.63	0.91	0.56	0.91	0.92
V1-61	0.61		0.40	0.51	0.51
V14-3	0.59	0.63	0.75	0.52	0.51
V1-59	0.54	0.00	1.14	0.33	0.41
V14-1	0.53	0.45	0.71	0.19	0.20
V9-4	0.53	0.43	0.41	0.10	0.10
V1-5	0.51	0.73	0.77	0.54	0.53
V1-42	0.49	0.94	1.09	0.55	0.54
V1-66	0.48	0.69	0.97	0.72	0.74
V1-54	0.47	0.71	1.11	0.27	0.28
V3-1	0.45	0.41	0.55	0.00	0.00
V2-4	0.39	0.19	0.18	0.28	0.26
V5-6	0.38		1.05	0.05	0.42
V3-5	0.33	0.14	0.06	0.22	0.22
V8-5	0.33	0.49	0.25	0.17	0.17
V9-2	0.32	0.46	0.45	0.18	0.19
V1-20	0.32	0.23	0.26	0.16	0.17
V1-85	0.31	0.49	0.78	0.59	0.59
V5-9-1	0.30	0.59	0.31	0.25	0.14
V1-47	0.29	0.27	0.41	0.00	0.00
V1-58	0.29	0.48	0.33	0.26	0.27
V1-84	0.26	0.32	0.36	0.25	0.25
V12-3	0.25	0.21	0.11	0.01	0.01
V13-2	0.25	0.28	0.22	0.03	0.03
V5-15	0.24	0.44	0.13	0.55	0.56
V1-63	0.24	0.42	0.48	0.15	0.15
V1-36	0.24	0.28	0.35	0.22	0.22
V1-11	0.24	0.25	0.06	0.09	0.09
V1-34	0.22		2.95	0.13	0.11
V1-77	0.19	0.30	0.40	0.24	0.23
V5-9	0.18	0.36	0.44	0.23	0.22
V5-12	0.18	0.26	0.26	0.18	0.18
V1-67	0.14	0.04	0.10	0.00	0.00
V11-1	0.12	0.01	0.00	0.00	0.00
V5-2	0.12	0.13	0.17	0.11	0.11
V5S21	0.11		0.31	0.02	0.14
V1-62-2	0.10	1.02	0.64		0.07
V1-71	0.10		0.64	0.13	0.07
V1-49	0.09	0.05	0.11	0.00	0.00
V3-4	0.08	0.08	0.11	0.00	0.00
V1-31	0.07	0.10	0.16	0.00	0.00
V15-2	0.07	0.06	0.11	0.03	0.03
V3-3	0.07	0.03	0.04	0.05	0.05
V7-4	0.07	0.13	0.08	0.20	0.20
V1S100	0.07				0.00

	KSU	Com1 (Orig)	Com1 (IMGT)	Com2 (Orig)	Com2 (IMGT)
V2-7	0.06	0.06	0.03		
V8-11	0.05	0.00	0.00	0.00	0.00
V1-43	0.05	0.03	0.05	0.00	0.00
V1S5	0.05	0.04	0.01	0.00	0.00
V1-23	0.04	0.03	0.19		
V1-37	0.04	0.03	0.06	0.02	0.02
V1-56	0.04	0.09	0.09	0.04	0.04
V3S7	0.03		0.00		
V8-4	0.03	0.01	0.01		
V8-6	0.02	0.00	0.00	0.00	0.00
V1-14	0.02	0.01	0.01	0.01	0.01
V6-7	0.02	0.00			
V1-62-3	0.02	0.15	0.15		0.34
V6-4	0.02	0.01	0.00	0.00	0.00
V1-62-1	0.01				
V5-12-4	0.01		0.00		
V1-17-1	0.01				
V6-5	0.01	0.00	0.00		

Supplemental Figure 1. Comparison of V-gene segment usage with original and KSU/IMGT bioinformatics. Com1 and Com2 V-gene segment usage with company specific bioinformatics (original) and after processing through the KSU bioinformatic pipeline (IMGT). The highest value percent of repertoire is dark read while the lowest are white. Black boxes represent no detected reads (true zero). Rounded zeros are represented as 0.0.
